# Symmetry in the front crawl stroke of different skill level of able-bodied and disabled swimmers

**DOI:** 10.1371/journal.pone.0229918

**Published:** 2020-03-19

**Authors:** Karini B. Santos, Paulo C. B. Bento, Carl Payton, André L. F. Rodacki

**Affiliations:** 1 Departament of Physical Education, Universidade Federal do Parana, Curitiba, Parana, Brazil; 2 Faculty of Physical Education, Universidade de Brasilia, Brasilia, Distrito Federal, Brazil; 3 Departament of Sport and Exercise Science, Manchester Metropolitan University, Manchester, England, United Kingdom; Universidade Federal de Mato Grosso do Sul, BRAZIL

## Abstract

Although swimming is recognized as a symmetrical sport, equivalence between each body side cannot be insured. Swimmers with physical and motor impairment may present asymmetries that are even more pronounced. Therefore, the objective of this study was to assess the symmetry of temporal coordination in the front crawl stroke phases and their dimensional characteristics among swimmers of different levels of skill and disabled swimmers. Forty-one swimmers (28 men and 13 women, 18,8 ± 3,3 years, divided 21 of them into groups of high and low level of skill and 20 in disabled swimmers group) performed a 50m maximum of front-crawl test while they were recorded by six synchronized cameras (four underwater and two above water) for analysis of the stroke phases, stroke dimensions (anteroposterior, mediolateral and vertical amplitude), index of coordination and hand speed. The symmetry index was calculated by the difference between the right and the left strokes. Comparisons were made using the Kruskal-Wallis test and multivariate comparisons were made using the Mann-Whitney test, with p <0.05. Asymmetry was noted in anteroposterior and mediolateral amplitudes of the stroke, index of coordination, duration of the recovery phase, each of the underwater phases and in the hand speed during the downseep phase, regardless of the level of skill or impairment. The disabled swimmers also showed asymmetry in the vertical amplitude of the stroke as well as in the insweep and upsweep speed. The reasons for these asymmetries may be the preference for unilateral breathing, force imbalance between pairs of homologous muscles and motor control deficit. The training with stereotypic movements may explain the similarity of asymmetries among the different groups of swimmers.

## Introduction

Front crawl swimming involves alternative bilateral coordination [1], consisting of a propulsive phase and a recovery stroke phase, which depend on the skill level to propel it forward and determine the swimming performance. Although swimming is apparently a symmetrical sport, with the front crawl style described as alternating stroke, the symmetry between the sides of the body is not guaranteed [2, 3]. In fact, asymmetries of kinetic [4, 5], arm coordination [3, 6] and shoulder roll and hip roll [7] have been observed, though conclusive consensus about their causality has not been reached yet. Reasons for asymmetry may be related to motor control deficit [8], arm dominance [2, 7] or factors associated with breathing technique and head position [9].

Previously studies have indicated three different possibilities of arm coordination adopted by swimmers during front crawl style. When there is a time lag between the propulsive phases of the two arms, the stroke coordination is called catch-up. When the start of the propulsive phases of one arm coincides with the end of the propulsive phase of the opposite arm, the stroke coordination is called opposition. When the propulsive phase of the left and right arms is overlapped, the coordination is called superposition [2].

The symmetry index (SI) is calculated to determine the coordination symmetry; negative results indicates asymmetry to the left side (e.g., greater than -10%), while positive results indicates asymmetry to the right side (e.g., greater than 10%) [9]. Small differences in strength between body sides are considered as acceptable and inherent to the human performance [10], however, differences greater than 10% are deemed as functional asymmetries [7, 11] that require compensatory strategies [11]. The same remark has been identified as the acceptable limit for the index of coordination [9, 12]. Even thought, the index of coordination (IdC) allows an assessment of the temporal symmetry in swimming [3, 8], this method disregards the recovery phase and the dimensional aspects of the stroke that may influence the temporal symmetries, that is, the anteroposterior amplitude, mediolateral amplitude and vertical amplitude of each stroke.

Asymmetries can be even more pronounced in disabled swimmers due to the very nature of their physical impairments. It is common, for instance, that they exhibit an asymmetrical anthropometric profile. In fact, Dingley et al. (2014) observed greater force asymmetry in disabled swimmers with more severe impairments. Investigations on symmetry are important to provide solid basis for compensatory training orientation aimed at preventing instabilities of the shoulder joint [2] and to respond to excessive asymmetries [6]. In addition, an adequate orientation can reduce the risk of premature fatigue by one of the body segments and may lead to a better performance [4, 13].

Therefore, the objective of this study was to assess the symmetry of temporal coordination in the stroke phases and their dimensional characteristics among swimmers of different levels of skill and disabled swimmers. It was hypothesized that the disabled swimmers would present the highest indexes of asymmetry, while the swimmers of high levels of skill would present the most symmetrical characteristics.

## Methods

Forty-one swimmers of both sexes participated in the study: 21 able-bodied swimmers (13 men and 8 women, 18.5 ± 3.78 years, males:1.79 ± 0.07 m; 71.50 ± 9.43 kg and females: 1.62 ± 0.06 m; 58.62 ± 7.17kg) and 20 swimmers with physical and motor impairments (15 men and 5 women, 19.35 ± 2.80 years, males: 1.70 ± 0.06 m, 61.49 ± 10.68 kg and female: 1.61 ± 0.12 m, 53.76 ± 12.58 kg). The inclusion criteria comprised: (i) minimum age of 15 years, (ii) minimum of a three-year competitive experience, and (iii) regular training frequency equals to, or more than, five times per week.

The disabled swimmers were classified according to the classes adopted by the International Paralympic Committee, from S5 to S10. Impairments included: amputation at the elbow level, cerebral palsy, myelomeningocele, arthrogryposis, double leg amputation at knee level, congenital malformation, dwarfism and spina bifida. Swimmers who had undergone recent joint surgery (less than 6 months) or brachial plexus palsy were excluded from the sample. Additionally, for the purpose of the present study, disabled swimmers with partial amputation or congenital malformation of the upper limbs were not included in the analysis of the asymmetry in the stroke dimensions (mediolateral amplitude, anteroposterior amplitude and vertical amplitude). The classification was performed at least 6 months before the data collection and resulted in 2 swimmers S5; 4 swimmers S6; 1 swimmer S7; 6 swimmers S8; 6 swimmers S9 and 2 swimmers S10.

Hence, for the analysis of temporal aspects of the movements, the group of was formed by 20 disabled swimmers (nine Brazilian and 11 British); and for the analysis of the dimensional aspects of the stroke, the group comprised 14 swimmers. The swimmers group was composed of 21 Brazilian swimmers, separated into 2 groups according to their skill level (technical index). They were allocated in a high skill level group (G1–11 swimmers) and in a low skill level group (G2–10 swimmers), according to the technical index proposed by the International Swimming Federation:

$$P=1000*\;{\left(B/T\right)}^{3},$$

where P refers to the score, B to the world record and T to the performance time of the swimmer. The average technical index of the swimmers of the high skill level group was 612 ± 93 points and of the low skill level group was 427 ± 66 points.

The swimmers were questioned about the breathing they usually use during training (unilateral or bilateral) and about arm lateral dominance. Four of the 41 swimmers reported that they use bilateral breathing (two G_2_ swimmers and two disabled swimmers). Regarding dominance, two swimmers from group G1 and four disabled swimmers reported that their left arm is dominant.

Participants were informed of the benefits and risks of the investigation. Minor participants' parents or guardians were also informed of the risks and benefits of the investigation. Written informed consent was obtained from all adult participants. Parents or guardians provided written informed consent for all participants 17 years of age or below. The full study protocol was approved by the Institutional Ethics Committee under the protocol number CAAE 30819514300000102.

### Instruments and procedures

Swimmers were invited to participate in a single experimental test held in a 25m length pool, which was covered and heated (~28°C). Anthropometric measurements (mass, height and arm span) preceded the experimental procedure. Prior to the swimming protocol, participants were familiarized (during the warm-up) with the instruments of data collection (swimming suites with attached LED markers). After 600 meters warm-up (freely at light to moderate intensity), swimmers were instructed to perform 50m maximum front crawl. In addition, they were asked not to breathe while passing through a pre-calibrated area, so that the breathing would not influence the movement. The start was performed inside the swimming pool and subjects received verbal encouragement during the test.

The 50m test was recorded by six video cameras, of which four captured videos underwater and two above water. The video cameras were synchronized by a light pulse, common to all of them. The video cameras used for the underwater recording of the Brazilian able-bodied and disabled swimmers were Gopro Hero 4 (60 Hz), while the British disabled swimmers were recorded by Sony HQ-DNR-1 (50Hz), connected to a video display device (GV-HD700). Sony HDR CX700 (30Hz) was used to record all subjects above water and only for checking, since only the movements of the underwater phase of the stroke would be reconstructed. Two video cameras were positioned diagonally on the right side of the swimmer and two on the left side, with approximate angles of 90° between each other. Each video camera focused on a space that had been previously calibrated in the swimming pool, with volume dimensions of 3.5 m (x) x 1 m (y) x 1.5 m (z) with 54 control points underwater and 36 points above water, from which the stroke data was collected.

Markers were positioned on the following anatomical landmarks: distal phalange of the third metacarpal and femoral trochanter in both right and left sides of the swimmers’ bodies. The markers used in the British disabled swimmers and in the Brazilian swimmers were spherical drawings of approximate 25mm of diameter, produced by a waterproof marker pen, while the markers used in the Brazilian disabled swimmers were LED light markers fixed on a suit made specially for this study. Further details to drawing markers and LED markers as well as their inter- and intra-operator reproducibility of measurements are described in Santos et al. (2017) and Dos Santos et al. (2017) respectively [14, 15].

The anatomical landmarks were digitized manually among the markers drawn on skin and the LED points were reconstructed semi-automatically from each video camera perspective by a specific software for kinematic analysis (SIMI Reality Motion Systems). The two-dimensional coordinates were filtered at 7 Hz using a Butterworth filter of low-pass (2nd order) and then converted into three-dimensional coordinates using a DLT algorithm [16]. One full stroke cycle–defined by the entry of one of the upper body segments into the water until the next entry of the same segment–was analyzed. The cycle was divided into 4 phases, adapted from Payton et al. (1999) [17]:

1. Downsweep: from the hand (or segment extremity for arm amputees swimmers) to entry into water to the most lateral position of the stroke.
2. Insweep: from the end of the first phase to the most medial position of the stroke.
3. Upsweep: from the end of the insweep to the hand exit of water.
4. Recovery phase: from the hand exit of water until its next entry.

**Fig 1:**
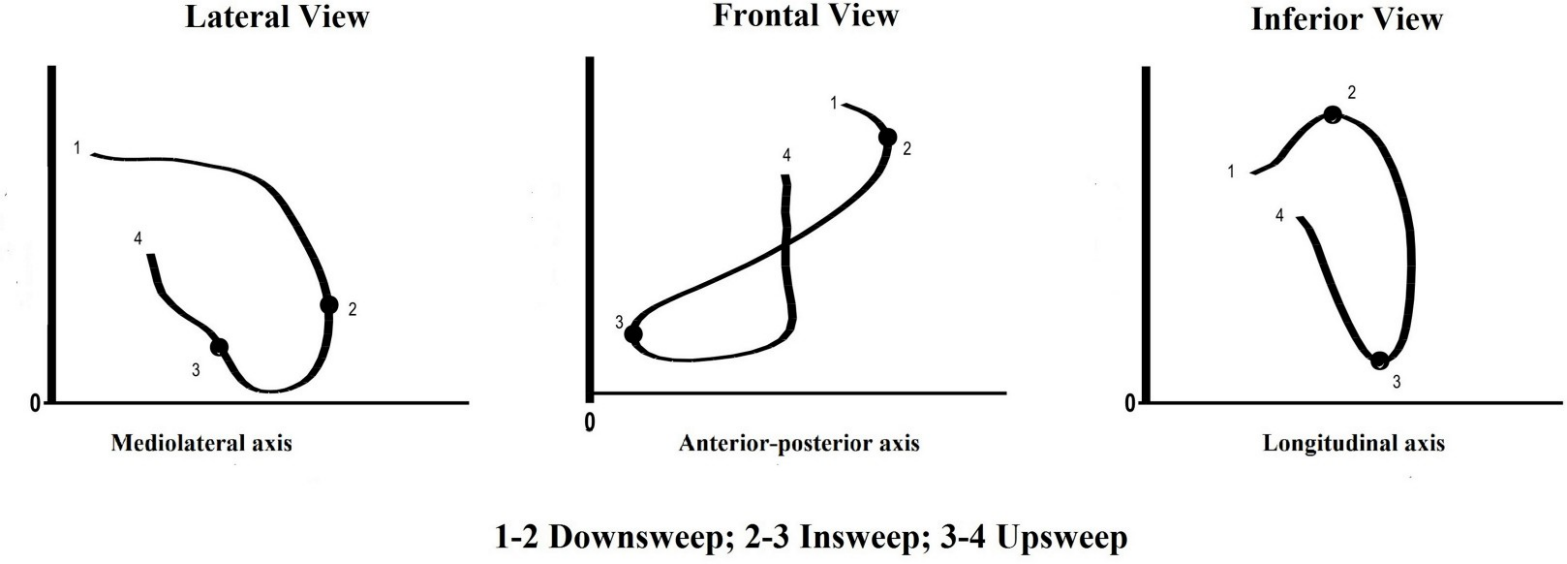
Example of the trajectory of the right arm of a swimmer (able-bodied) and its phase divisions.

The sum of the first three phases (downsweep, insweep and upsweep) corresponds to the underwater phase. Fig 1 shows a typical stroke trajectory from one participant, with indication of the underwater stroke phases.

The following kinematic variables were used to describe the swimming movements:

- Anteroposterior amplitude of the underwater stroke: dislocation in the x-axis by the difference between the positions of hand entry and hand exit during underwater phase.
- Mediolateral amplitude of the underwater stroke: dislocation of the y-axis by the difference between the highest lateral position and highest medial position.
- Vertical amplitude of the stroke: dislocation of the z-axis between the hand entry into water to the deepest point.
- Stroke trajectory: result of the stroke amplitudes (mediolateral, vertical and and anteroposterior).
- Percentage of time spent in the underwater phase: percentage time between the hand entry into water and its exit in relation to the time of the full stroke cycle.
- Index of Coordination (IdC): adapted from Chollet et al. (2000), considering opposition between strokes (IdC = 0), catch-up (IdC < 1) or arm superposition (IdC> 1) in the propulsive phase (insweep + upsweep corresponding to pull and push phases).
- Mean hand speed in the underwater phase: ratio between the trajectory resulting from the underwater phase (i.e. resulting from all axis) and the time spent to complete the underwater phase.
- Mean hand speed in each underwater phase of the stroke: the ratio between the trajectory in each underwater phase (i.e., downsweep speed, insweep speed and upsweep peed) and the time spent to complete each phase.

In order to determine the bilateral symmetry, the symmetry index (SI) was estimated by the absolute value of an adaptation of the calculation proposed by Robinson et al. [18].

$$SI=\left(\frac{{(VARIABLE}_{RIGHT}+{VARIABLE}_{LEFT})}{{0.5\;(VARIABLE}_{RIGHT}+{VARIABLE}_{LEFT})}\right)100$$

Differences in percentage lower than 10% were indicated as bilateral symmetry while those higher than 10% as asymmetry [10, 13].

### Statistics

Since the majority of data had not followed a normality pattern, the comparisons of the asymmetry in the dimensional and temporal variables among the groups were made using the Kruskal-Wallis nonparametric test. For the multivariate comparisons (post hoc), the Mann-Whitney test was used, with the p-value divided by the number of comparisons made (3). The significance level was set at p<0.05 and the analyses were performed using the Statistica software, version 7, Statsoft.

## Results

**Fig 2:**
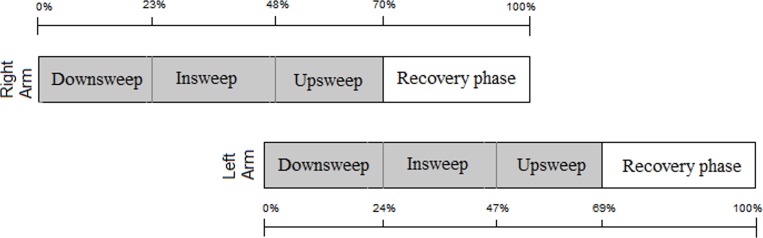
Representation of the stroke phases for the right and left sides of the swimmers assessed (41).

Fig 2 represents the average percentage of time of all swimmers (G1 _+_ G2 _+_ G3) to complete each stroke phase for the right and left sides.

Asymmetry between sides was observed in the three groups in anteroposterior and mediolateral amplitude of the underwater stroke, index of coordination, percentage of time spent in the recovery phase and in each of the underwater phases, as well as in the speed during the downsweep phase. In addition, the disabled swimmers showed asymmetry in the vertical amplitude of the stroke and speed during the insweep and the upsweep. During the insweep, the asymmetry index for hand speed of the disabled swimmers group did not differ from the other groups. The groups’ percentage for each variable are shown in Table 1.

**Table 1:** Comparison of the percentage of asymmetry in the swimming variables among groups of high skill level (G1), low skill level (G2) and disabled swimmers (G3).

	**G1 (11)**	**G2 (10)**	**G3 (14)**	**p**
**Anteroposterior amplitude**	17.4* (9.50–25.00)	10.49* (7.37–15.44)	14.82* (12.63–19.81)	0.24
**Mediolateral amplitude**	27.05* (20.60–32.53)	26.01* (16.01–35.16)	37.43* (23.06–42.55)	0.22
**Vertical amplitude**	6.78 (3.57–7.91)	9.68 (6.69–11.60)	9.31 (4.24–14.36)	0.22
**Stroke trajectory**	8.26 (3.90–11.35)	4.65 (2.12–9.67)	3.22 (1.42–13.83)	0.32
	**G1 (11)**	**G2 (10)**	**G3 (20)**	**p**
**Underwater phase**	6.99 (1.74–9.625)	2.74 (2.47–8.19)	5.76 4.25–8.72	0.42
**Recovery phase**	15.28* (3.22–23.01)	11.57* (4.39–18.80)	14.90* 9.17–21.00	0.42
**Index of coordination**	52.63* (28.57–166.67)	60.00* (33.33–88.89)	88.37* 58.82–166.66	0.57
**Downsweep**	16.64* (9.66–42.67)	30.75* (17.22–41.05)	23.14* 11.43–46.21	0.71
**Insweep**	19.88* (7.32–27.05)	13.36* (5.97–23.03)	15.64* 6.45–24.82	0.84
**Upsweep**	16.31* (5.63–37.925)	15.58* (11.65–33.11)	21.64* 10.39–69.48	0.63
**Downsweep Speed**	15.91* (5.58–20.37)	15.46* (9.00–25.74)	12.36* 2.86–24.89	0.45
**Insweep Speed**	3.86 (1.12–16.16)	5.15 (1.53–7.86)	11.89*^a^ 9.390–28.25	0.04
**Upsweep Speed**	9.96 (5.97–13.51)	8.22 (7.32–11.76)	11.88* 5.45–22.63	0.59
**Underwater phase Speed**	5.79 (0.83–9.13)	4.76 (3.06–6.29)	6.13 2.56–10.01	0.64

Values are presented as medians and interquatile ranges (1^st^ and 3^rd^).

*Bilateral asymmetry.

^a^ difference in relation to G1. p—comparison between groups asymmetries

## Discussion

This study aimed to determine the symmetry in the dimensional characteristics of the front crawl stroke, temporal coordination and hand speed in swimmers of low and high skill levels and disabled swimmers. The main finding of the study is that able-bodied (regardless of skill level) and disabled swimmers present bilateral asymmetries. To our knowledge, this is the first study that assessed asymmetry in multiple three-dimensional swimming variables in groups of able-bodied swimmers and disabled swimmers. The average time spent by the groups in each underwater phase of the stroke showed a similar percentage of time (i.e. 22 to 25%) and the underwater phase represented more than two thirds of the stroke, which is relevant aspect, as the propulsion occurs at theses phases. In addition, an apparent coordination symmetry was established (Fig 2). This refers to the fact that some participants presented asymmetries in one direction and others in the opposite direction. Thus, the asymmetries disappeared when the group average was determined. However, when the symmetry index was calculated for each subject, without considering its direction (right or left), asymmetries were detected (Table 1).

Since more than 90% of the swimmers were right-handed, and asymmetries were found in both directions, the lateral dominance does not appear to be a factor that explains this result. If the dominance were responsible for the asymmetries, most of the participants (19 swimmers) would show difference in the same direction and the average would reflect these asymmetries. It is noteworthy that despite the asymmetries between the anteroposterior and mediolateral amplitudes of the stroke, the total trajectory presented the same amplitude. It seems that the swimmers compensate for the mediolateral differences with the anteroposterior differences and vice versa, in order to generate amplitudes of equivalent trajectories between strokes.

The index of coordination for the arms was the variable that presented the largest asymmetries. Tourny-Chollet et al. (2009) also verified a high asymmetry index in coordination with high variability among the swimmers (25 to 170%). This leads us to think that a motor control deficit may explain the lateral differences observed, or that different functions between the body segments are employed. There are arguments that one arm may be responsible for the production of higher levels of strength and the other one responsible for actions related to control and support [19, 20]. However, it is necessary to take into account the limitation inherent to index of coordination, as it does not consider the kicking propulsion.

The differences between the index of coordination for the arms have been previously described in the literature and explained by motor control deficits [8], dominance [2], breathing [2, 6], and asymmetrical body roll [7]. In the present study we assessed the stroke disregarding the breathing, and yet asymmetries were observed. Therefore, breathing cannot be pointed out as the only cause for the discrepancy between contralateral strokes. However, less than 10% of the subjects reported use of bilateral breathing, which indicates that possible asymmetries may be generated from the repeated use of unilateral breathing. In addition, the asymmetries were determined without considering the side (left or right), therefore, the asymmetries in the present study cannot be explained by factors related to dominance. Possible differences in the capacity of strength application between strokes can partially explain the bilateral differences. The use of compensatory movements in the stroke may have been employed in order to create a more symmetrical strength application [11].

When the stroke was divided into underwater and aerial phases, the swimmers presented asymmetry between sides in the percentage of time spent in the recovery phase, and symmetry in the underwater phase. Barden et al. (2011) reported asymmetries between the right and left side in the underwater and aerial phases, with larger differences in the underwater phase [21]. The contrast between the present study and the results found by Barden et al. (2011) can be explained by the fact that the authors used absolute values, while the present study analyzed the relative asymmetry in each phase, since they present a distinct percentage of duration (~30% versus ~70% for the aerial and underwater phases, respectively) and when considering absolute values, such relations can be overestimated or underestimated.

Although the underwater phase is equivalent between the right and left side for the percentage of time spent, when the new subdivisions were analyzed, that is, considering the stroke sweeps (down, in and up), asymmetries were identified in all phases for all groups. It seems that for the same total stroke duration swimmers present different strategies to organize each phase. One plausible explanation for such differences could be the changes in the swimming technique to allow breathing, however, as subjects were requested not to breath while passing in the data collection area, this argument can be ruled out. On the other hand, it may have occurred due to individual strategies that require further investigation.

The hypothesis that the skill level influences the symmetry between the movements performed by the upper body segments was not confirmed, since there were no difference between the groups of high and low skill levels. These data are corroborated by Psycharakis and Sanders (2010), who did not find correlation between bilateral asymmetries and swimming speed [22]. In addition, Morouço et al. (2015) did not find association between strength asymmetry and the swimming skill level. It seems that bilateral symmetries are not able to differentiate the skill level of the swimmers. Indeed, swimmers can purposefully employ individualized standard technique for each stroke, attributing it to a segment more focused on the production of upper strength, and to another focused on actions more related to control and support.

Similarly, the asymmetry found in the disabled swimmers did not differ from the other groups, with the exception of the speed asymmetry in the insweep phase, which was larger than that observed in the group of swimmers of low skill level. Moreover, the upsweep speed was asymmetrical only in the group of disabled swimmers. Thus, the hypothesis that the disabled swimmers would have the largest asymmetries was partially confirmed. Perhaps the movements performed (and trained) during swimming are stereotypic and correspond partly to a similar technique observed in the able-bodied swimmers. The repetitive movements of swimming may usually involve the learning of a stereotypic movement technique [4]. Moreover, disabled swimmers with partial upper limb amputation were not included in the dimensional analyses of the stroke, which ensured greater homogeneity of the data and resulted in a sample largely composed by swimmers without upper limb impairments (or similar, ex., dwarfism).

Higher levels of strength asymmetry in disabled swimmers have been observed in swimmers with more severe impairments [23]. Due to the reduced number of swimmers allocated in each of the functional classes, we decided not to compare sub-groups and take into consideration the general behavior of the disabled swimmers assessed (S5-S10). Despite the asymmetries identified in the speed of the insweep and upsweep phases, when the underwater stroke was considered as a whole, the speed was symmetrical, which indicates that compensation is performed to result in a speed of stroke trajectory equivalent between sides.

This study presented results of symmetry/asymmetry among the averages obtained by selected groups (swimmers of high and low skill levels and disabled swimmers of classification S5-S10). Perhaps the movements trained during swimming are stereotypic and correspond to a similar technique between them. However, the individual behavior of the subject within a group was not considered, which represents a limitation of the study. Indeed, the small number of disabled swimmers in each functional classification did not allow multivariate analysis by groups. Lastly, different frequency of images acquisition (60 and 50 Hz) used in Brazilian and British para-swimmers could affect results comparisons. Further studies including individual analysis are warranted.

## Conclusion

The results of this study provide swimmers and coaches with an overview regarding symmetries in front crawl stroke between different skill level of swimmers and disabled swimmers. The swimmers showed asymmetry in anteroposterior and mediolateral amplitudes of the stroke, index of coordination, subdivisions of the phases (downsweep, insweep, upsweep and recovery) and speed in the first phase of the underwater stroke, regardless of the skill level or impairment. There was no difference in the symmetries/asymmetries among the groups, with the exception of the upsweep speed, which was different between disabled swimmers and low skill level swimmers. The preference for unilateral breathing, strength imbalance between pairs of homologous muscles and motor control deficit are possible explanations for the swimmers asymmetries. However, the high variability of results indicates that subjects differ among themselves and that intervention with compensatory training for the reduction of asymmetries is recommended only after the analysis of the swimmer’s individual response.
